# Bioactive Phytochemicals of *Acacia saligna*

**DOI:** 10.3390/molecules28114396

**Published:** 2023-05-28

**Authors:** Alison T. Ung, Anjar P. Asmara

**Affiliations:** School of Mathematical and Physical Sciences, Faculty of Science, University of Technology Sydney, Ultimo, NSW 2007, Australia; anjarpurba.asmara@student.uts.edu.au

**Keywords:** *Acacia saligna*, hydroxybenzoic acids, cinnamic acids, flavonoids, antioxidant, antimicrobial, anticancer, α-glucosidase inhibition, anti-inflammation, structure–activity relationship

## Abstract

*Acacia saligna* is native to Western Australia. It has become an introduced and fast-growing plant in other parts of the world due to its ability to adapt to drought, saline and alkaline soils, and hast growing environments. Studies on the bioactivities and phytochemicals of the plant extracts were conducted. However, comprehensive information that links those bioactivities to the identified compounds in the plant’s extracts is still lacking. Data gathered in this review revealed a rich chemical diversity of hydroxybenzoic acids, cinnamic acids, flavonoids, saponins, and pinitols in *A. saligna* growing in Egypt, Saudi Arabia, Tunisia, South Africa, and Australia. The variability in phytochemical composition and quantity could be attributed to plant parts, growing locations, extraction solvents, and analysis methods. Identified phytochemicals support observed biological activities such as antioxidant, antimicrobial, anticancer, α-glucosidase inhibition, and anti-inflammation in the extracts. The knowledge of chemical structures, biological activities, and possible mechanisms of action of the bioactive phytochemicals identified in *A. saligna* were discussed. In addition, the structure–activity relationships of dominant active compounds were examined to explain the bioactivities exerted by *A. saligna* extracts. The review provides valuable insights towards future research and the development of new therapeutics from this plant.

## 1. Introduction

There are about 1380 species of *Acacia* worldwide, and about two-thirds of them are native to Australia [1]. Indigenous Australians have used the plants’ leaves, bark, and flowers as medicinal agents for centuries [2]. Decoction and infusion have been the most common preparations for ethnomedicinal plants [3,4,5]. The Australian continent consists of some arid, semi-arid, and dry subtropical regions, which allow Acacia plants to grow and produce unique secondary metabolites [6]. In addition to their survival purpose, the phytochemicals have various benefits for human health, such as anti-digestive disorders (tannins, saponins, flavonoids); anti-plasmodial (tryptamine, tannins, organic acids, saponins); antioxidant (polyphenols); anticancer (triterpenoids, saponins); nutraceutical, diuretic and natriuretic therapies (polysaccharide, glucosides, and gum) [7]. The Acacia plants of medicinal importance are *A. kempeana, A. ligulata, A. tetragonophylla, A. mearnsii,* and *A. pycantha*. They have been reported to contain rich contents of polyphenolic compounds in their extracts. Another Acacia of note is *A. saligna*. *A. saligna* is commonly found on poor sandy soils and coastal dune systems. The tree is a highly tolerant species against drought, saline and alkaline soils, and frosty environments [8]. Due to its ability to stabilise a coastal dune system, this species was cultivated in the Eastern States of Australia and some countries in the Middle East, Africa, and South America. Although it is not known to be used as traditional medicine, *A. saligna* has been studied for its in vitro bioactivities and phytochemicals from different plant parts growing in the Middle East, Africa, and South America. However, investigations into the biological activities and phytochemicals of the plant growing in Australia have been limited.

### 1.1. Acacia Saligna 

*Acacia saligna* (Labill.) H. L. Wendl. (1820) is the current scientific name for the species characterised by 2–10 m tall shrubs or small trees, which have grey to red-brown bark, are linear to the lanceolate; measure 8–25 × 0.4–2 cm; have green to glaucous leaves, and have bright, yellow-rounded flowers that measure 5–10 mm in diameter. The flowering season is usually between August and October, while mature legumes appear from November to January. It is native to Western Australia and was previously named *A. cyanophylla* Lindl, *A. bracteate* Maiden and Blakeley, *A. lindleyi* Meissner, *Mimosa saligna* Labill., and *Racosperma salignum* (Labill.) Pedley [9]. The plant is also recognised locally as the Port Jackson wattle, Coojong, blue-leafed wattle, and Western Australia golden wattle. The following information in Table 1 shows a detailed taxonomic chart of the species taken from Maslin [10]. 

Most investigations have been dedicated to screening extracts of *A. saligna* growing in Saudi Arabia, Egypt, Tunisia, and other parts of Africa against various biological targets. These investigations have shown that the extracts possessed various bioactive phytochemicals. For instance, flavonoids isolated from the flowers and leaves have had antifungal, antioxidant, anti-acetylcholinesterase, and antibacterial activities [11,12,13].

The volatile phytochemicals of the flowers possess allelopathic activity, thus indicating their potential as bioherbicides [14,15]. The isolated compounds from these extracts have been shown to have antioxidant activities and cytotoxicity against liver cancer cells [16,17]. Ethanolic crude extracts from the plant’s bark show antifungal and antioxidant activities [18], as well as α-glucosidase inhibitory activity [19]. In the Middle East, Africa, and South America, *A. saligna’s* parts have been used as animal feeds [20,21,22], thus suggesting this plant’s low toxicity and high nutritional benefits.

### 1.2. Aims

Currently, no comprehensive review links the chemistry, biology, or medicinal aspects of the phytochemicals in *A. saligna*. This review is not exhaustive in its coverage of all phytochemicals found in *A. saligna* growing in Egypt, Saudi Arabia, Tunisia, South Africa, and Australia. Instead, it aims to focus on the compounds that show biological activities with potential applications as pharmacological drugs that are urgently needed for human health to combat infectious diseases (antimicrobial resistance: methicillin-resistant *Staphylococcus aureus (MRSA), Escherichia coli, Klebsiella pneumoniae*, and *Mycobacterium tuberculosis*) [23] and noncommunicable diseases (Type 2 diabetes, cardiovascular diseases, cancers, and chronic inflammatory diseases) [24]. This review also aims to broadly examine the compositions and contents of the bioactive compounds in *A. saligna* from different parts of the plant that yield under the influences of the growing environment, different cultivation locations, and extraction solvents. The compositions and contents of these compounds provide a link to the observed biological activities. We also highlighted the structure–activity relationships (SAR) and mechanisms of action (MOA) of the phytochemicals identified in *A. saligna*. 

To date, no review has been created on the phytochemicals and bioactivities of the extracts or isolated compounds from *A. saligna*. Therefore, this review can guide further studies on this plant to develop optimised methods for isolating, identifying, and testing bioactive extracts and isolated compounds. SAR and MOA information can assist in drug design and discovery from natural products.

## 2. Phytochemicals from *A. saligna*

Our literature survey has revealed that the phytochemical analyses of *A. saligna* were mainly conducted using plant materials growing in Saudi Arabia, Egypt, Tunisia, South Africa, and, recently, in Australia. Several reported analytical methods were employed to identify and quantify compounds in the plant extracts. For instance, classical and low-cost colorimetric methods were most used by authors to proximate the total phenolic and flavonoid contents in the extract. The total phenolic content was evaluated using the standard Folin–Ciocâlteu reagent with gallic acid (GA), while the flavonoid content was determined using the standard aluminium trichloride method with rutin. High-performance liquid chromatography–photodiode array detection (HPLC-DAD), as well as HPLC with variable wavelength detector (VWD) were used. Authentic standard retention times and calibration curves were used in the HPLC-based analyses to provide a qualitative and quantitative analysis of compounds in the extracts. Single compound isolation and structure elucidation using NMR techniques were also employed. Gas chromatography coupled with mass spectrometry (GC-MS) analysis was mainly used to determine the volatile components of the extracts. The sections below describe the phytochemicals found in the individual parts of the plant. The composition and quantity of identified compounds from each crude extract are listed in Supplementary Table S1, which are related to the plant parts, extraction solvents, analysis methods, and harvesting locations. The chemical composition and content in each active extract related to their bioactivities will be discussed in detail in Section 3.

### 2.1. Phytochemicals from Flowers

**Figure 1:**
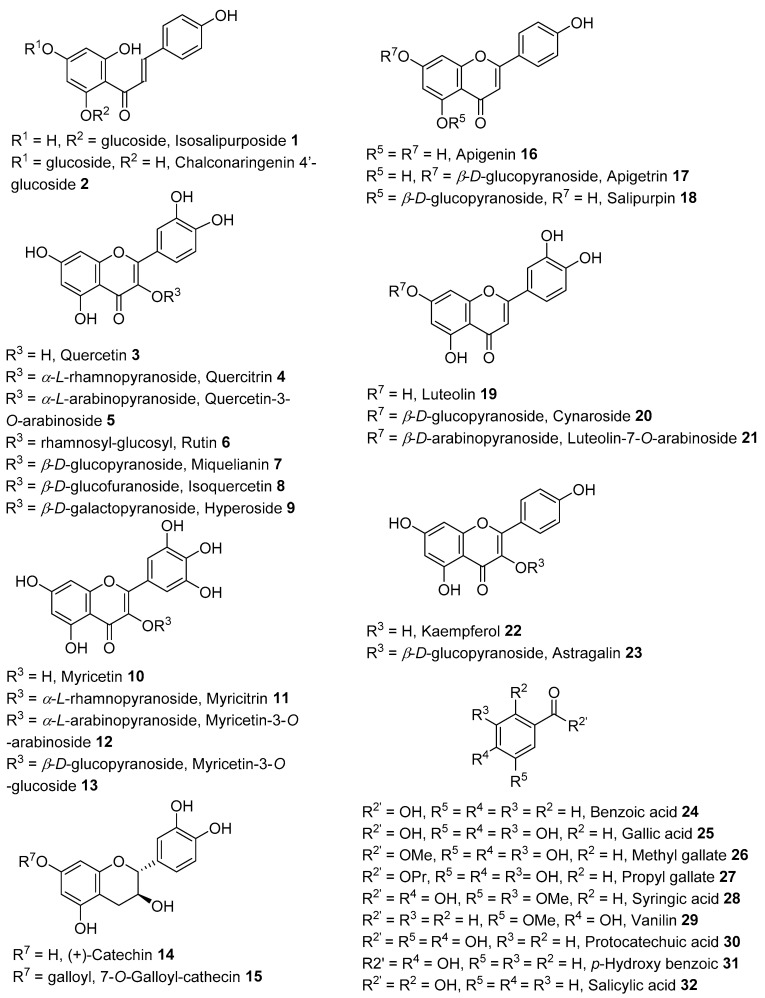
Phenolic and flavonoid derivatives identified in flowers [11,12,26], leaves [13,16,25,26,29], and the bark [18] of *A. saligna*.

The literature has revealed that flavonoid derivatives were the most common phytochemicals reported from the flowers of *A. saligna,* along with beneficial effects such as antioxidant compounds [11,12,25]. Isosalipurposide **1**, quercetin **3** and naringenin **42** (Figure 1) have been isolated from the ethyl acetate extract of flowers harvested in Tunisia by Ghribia et al. [11]. An HPLC-based study conducted by Al-Huqail et al. [12] revealed the presence of quercetin **3**, kaempferol **22**, benzoic acid **24**, syringic acid **28**, *p*-hydroxybenzoic acid **31**, salicylic acid **32**, caffeic acid **35**, *o*-coumaric acid **36**, *p*-coumaric acid **37**, ferulic acid **38**, naringenin **42**, ellagic acid **44**, catechol **45**, and caffeine **46** in the water-soluble extract of flowers harvested in Egypt. 

**Figure 2:**
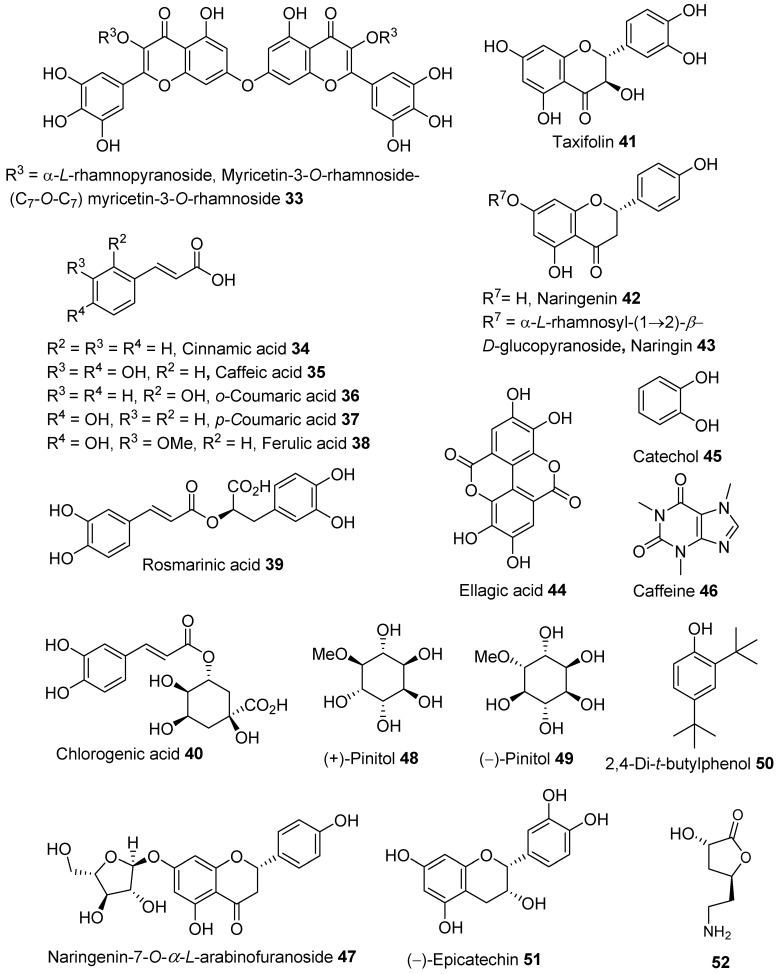
Phenolic and flavonoid derivatives and other compounds identified in flowers [11,12,26], leaves [13,16,25,26,29] and the bark [18,26] of *A. saligna*.

Recent work by our group [26] on Australian *A. saligna* showed that the methanolic extract of the flowers obtained via sequential solvent polarity extraction was bioactive. A column chromatography separation of the extract provided naringenin **42**, isosalipurposide **1**, quercitrin **4** and *D-(+)*-pinitol **48**, and naringenin-7-*O*-*α*-*L*-arabinofuranoside **47**. Quercitrin **4** was the main compound, followed by naringenin-7-*O*-*α*-*L*-arabinofuranoside **47** and *D-(+)*-pinitol **48** (Figure 2). The latter two compounds were recently isolated from *A. saligna* growing in Australia. 

**Figure 3:**
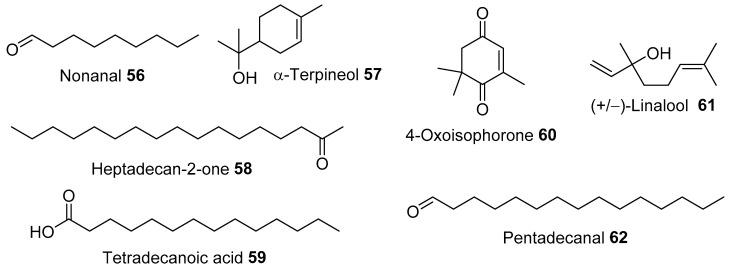
Some volatiles from the flowers and leaves of *A. saligna* identified by GC/CI-MS analysis [14].

Gas chromatography with flame ionisation detection (GC-FID) and GC-MS analysis of the flowers were collected in Tunisia by El Ayeb-Zakhama et al. [14], which revealed 16 volatiles. Among these, nonanal **56** was the major compound, which contained 66.5% *w*/*w* of the total essential oils. *α*-Terpineol **57** (8.4%), heptadecan-2-one **58** (5.8%), and tetradecanoic acid **59** (5%) were other important components (Figure 3). 

### 2.2. Phytochemicals from Leaves

Some flavonoid derivatives such as astragalin **23** [27], catechin derivatives (**14**, **15**), quercetin derivatives (**3**–**5**), myricetin derivatives (**10**–**13**), rutin **6**, naringenin **42**, taxifolin **41**, and luteolin derivatives (**19**–**21**) were identified in leaves collected in Egypt [25,28] and Saudi Arabia [13,17], as shown in Figure 1. HPLC-based studies conducted by Guneidy et al. [28], Elansary et al. [13], and Gumgumjee et al. [17] reported that crude extracts from leaves contained rich compositions of benzoic acid derivatives such as gallic acid **25**, syringic acid **28**, vanillin **29**, protocatechuic acid **30**, and *p*-hydroxybenzoic **31** (Figure 1). Of note were cinnamic acid **34**, caffeic acid **35**, *p*-coumaric acid **37**, ferulic acid **38**, chlorogenic acid **40**, and other phenolic esters, including methyl gallate **26**, propyl gallate **27,** and ellagic acid **44**. 

The sequential solvent polarity extraction of leaves collected in Australia provided a bioactive methanolic extract. Upon two successive purifications via column chromatography of this extract, we obtained (–)-epicatechin **51**, quercitrin **4**, myricitrin **11**, 2,4-di-*t*-butylphenol, (−)-pinitol **49**, and (3*S,*5*S*)-3-hydroxy-5-(2-aminoethyl)-dihydrofuran-2(3H)-one **52**. The latter three compounds were the first to be isolated from *A. saligna* growing in Australia and in other parts of the world [26].

**Figure 4:**
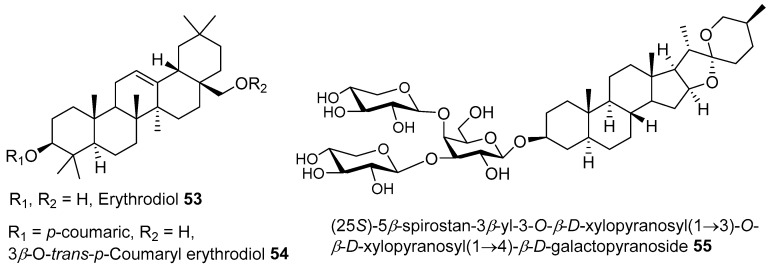
Saponin derivatives isolated from methanolic extract of *A. saligna* leaves collected in Tunisia [25].

Derivatives of saponin (**53**–**55)** (Figure 4) were also identified, along with the biflavonoids glycoside **33**, quercitrin **4,** and myricitrin **11,** in methanolic leaf extracts by Gedara et al. [25].

Gas chromatographic analyses (GC-FID and GC-MS) of volatiles from the leaves collected in Tunisia identified 21 compounds (Figure 4). Among these (reported in % of the total content), nonanal **56** (22.8), *α*-terpineol **57** (12.1), 4-oxoisophorone **60** (11.5), (+/−)-linalool **61** (9.4), heptadecan-2-one **58** (8.8), and pentadecanal **62** (4.7) were detected in reasonable quantities [14].

### 2.3. Phytochemicals from Barks 

HPLC-based analysis of an ethanolic crude extract of *A. saligna* bark collected in Egypt [18] revealed the presence of naringenin **42**, rutin **6**, kaempferol **22**, benzoic acid **27**, gallic acid **28**, chlorogenic acid **41**, vanillin acid **36**, caffeic acid **38**, ferulic acid **40**, rosmarinic acid **41**, and caffeine **43** (Figure 1 and Figure 2). This ethanolic extract was screened for antioxidant and antifungal activities. Buttner et al. reported the phenolic content of an ethanolic bark extract from *A. saligna* harvested in South Africa using the Folin–Ciocâlteu reagent analysis [19]. The compounds (−)-epicatechin **51**, *D-*(+)-pinitol **48** (Figure 2), and sucrose were isolated from a bioactive methanolic bark extract of Australian *A. saligna* [26].

### 2.4. Variability in Phytochemical Compositions of A. saligna

Plants biosynthesise secondary metabolites for survival and defence. The outcome of biosynthesis is influenced by environmental factors, including soil, salinity, temperature, and geographical variations [30]. Environmental stresses, such as drought and high salinity, can stimulate the signalling pathway in the plant’s cells to accumulate bioactive compounds in order to respond to severe conditions. For instance, *D*-pinitol **48** (methoxy inositol) was found in Australian *A. saligna* [26]. This suggested that plants grown in water shortage and high salinity areas produced the compounds as osmoprotectants to overcome the osmotic stress. A study by Streeter et al. [31] confirmed that *D*-pinitol **48** (methoxy inositol) in stressed soybean leaves was around 40% higher in concentration than in the well-watered groups. 

Moreover, studies on drought and salinity stresses showed significantly elevated levels of gallic acid **25** and *p*-coumaric acid **37** in *A. saligna*. For example, the leaves harvested from the Albahah region of Southwest Saudi Arabia yielded (reported in % of the total content) gallic acid **25** (0.0054) and *p*-coumaric acid **37** (0.0008) [17]. In contrast, the leaves collected from the Orman Botanical Garden in Giza, Egypt yielded a much higher content of gallic acid **25** (19.2) and *p*-coumaric acid **37** (6.4) [29]. The Albahah region is a dry area with an annual mean rainfall of 142.6 mm/year [32] and a medium to high soil salinity [33], while the Giza site is an arid area estimated to have 1.2 mm/year of annual rainfall [34] and a high soil salinity [35]. These findings suggest the influences of abiotic stresses in phenolic biosynthesis.

Furthermore, plants have been known to produce high concentrations of flavonoids and glycosides when they experience environmental stresses such as low or high temperatures, high intensity of light, high salinity, and drought [36]. These flavonoids were identified (with variable quantities) in the extracts from various parts of *A. saligna* plants from Egypt, Saudi Arabia, Tunisia, South Africa, and Australia, wherein the plants shared similar growing conditions and environments.

Our data in Supplementary Table S1 revealed variations in the quantities and types of polyphenolic compounds. It is noteworthy that three main groups (flavonoids, cinnamic acids, and benzoic acids) of compounds were commonly identified (with variable quantities) across the plant parts, geographic origins (growing conditions), and polarity of the extraction solvents and analytic methods. Although most articles did not mention the collection period and phenological stage, the abiotic factors could influence the extracts’ chemical composition outcomes. These commonly identified compounds (with variable concentrations) across actives extracts (Supplementary Table S1, Figure 1 and Figure 2) are

(i) flavonoids—isosalipurposide **1,** quercetin **3**, kaempferol **22**, rutin **6**, and naringenin **42**;
(ii) hydroxycinnamic acids—caffeic acid **35**, *o*-coumaric acid **36**, *p*-coumaric acid **37**, and ferulic acid **38**; and
(iii) benzoic acid **24** and hydroxybenzoic acids such as gallic acid **25,** salicylic acid **32,** ellagic acid **44,** syringic acid **28**, and *p*-hydroxybenzoic acid **31**.

In summary, richer chemical diversity has been observed among compounds in the leaf and flower extracts than in bark extracts. The differences in the chemical compositions of *A. saligna* crude extracts can influence the outcomes of their biological activities [37]. 

## 3. Bioactivities of *A. saligna* Extracts and Identified Phytochemicals

In the following sections, we will discuss the biological activities of *A. saligna* extracts that are important for human health. The bioactivities, including the possible MOA and SAR of the identified compounds, will be discussed to justify the bioactivities exerted by the crude extracts. The SAR described for a specific target can guide the design and synthesis of derivatives with the structural features required for activity.

### 3.1. Antioxidant 

The literature indicates that the antioxidant properties of *A. saligna* crude extracts and isolated compounds have been the most reported compared to other bioactivities due to the accessibility [38], practicality, and low cost of the assays. Two commonly used assays are in vitro scavenging free radicals 2,2-diphenyl-1-picrylhydrazyl (DPPH), and 2,2′-azino-bis-(3-ethylbenzothiazoline-6-sulphonic acid) (ABTS). DPPH is a stable free radical that readily accepts an electron or a hydrogen radical from a reducing agent such as vitamin C (ascorbic acid). The reaction results in the formation of stable dehydroascorbate and DPPH-H [39]. DPPH exhibits a strong absorption band in a methanolic solution with a deep purple colour. The reduction of DPPH radicals has been routinely monitored spectrophotometrically at 517 nm. In those experiments, the colour of the solution gradually decreased as the concentration of the antioxidant increased, wherein DPPH radicals converted to the colourless DPPH-H. 

The antioxidant activities in this discussion were obtained using the DPPH method, wherein vitamin C or butylated hydroxytoluene (BHT) was used as a positive control. The plant parts of *A. saligna* were mainly harvested in Egypt, Tunisia, Saudi Arabia, and Australia. The active extracts were found to be those of the alcoholic extracts. The alcoholic extracts from the bark, leaves, and flowers were shown to have substantial antioxidant activities, which can be ranked from barks> leaves > flowers based on the IC_50_ values of the extracts. HPLC-based analysis and bioactive-guided fractionation of the plant extracts revealed polyphenolic and flavonoid derivatives, as shown in Table 2. 

Ghribia et al. [11] performed sequential polarity-based extraction of a methanolic crude extract of *A. saligna* flowers collected in Tunisia (Table 2). Dichloromethane, ethyl acetate, *n*-butanol, and aqueous extracts were obtained. Among these four extracts, the ethyl acetate extract was most active in the DPPH assay, with an IC_50_ of 67.26 μg/mL. The ethyl acetate extract was subjected to pure compound isolation using column chromatography, which yielded (reported in % *w*/*w* of the dried flowers) isosalipurposide **1** (0.75), quercetin **3** (0.002), and naringenin **42** (trace amounts). Isolated quercetin **3** was screened in the same DPPH assay and was found to be most active, with an IC_50_ of 4.58 μg/mL, which was comparable to the positive control reference quercetin’s IC_50_ of 4.77 μg/mL. Isosalipurposide **1** was the second most active compound, with an IC_50_ of 81.9 μg/mL, which was three times more active than naringenin **42** (255.5 μg/mL). The activities of isosalipurposide **1** and quercetin **3** support the potent antioxidant activity exerted by the ethyl acetate extract.

The aqueous crude extract of flowers collected in Alexandria, Egypt by Al-Huqail et al. [12] was found to inhibit DPPH with a much weaker activity (IC_50_ = 463.71 μg/mL) compared to that reported by Ghribia et al. [11]. The HPLC-based analysis of the aqueous extract from the flowers revealed a rich composition of benzoic acids, cinnamic acids, and flavonoids (Table 2). Among those identified were (reported in % *w*/*w* of crude extract) were quercetin **3** (0.112), kaempferol **22** (0.0445), naringenin **42** (0.145), syringic acid **28** (0.006), *p*-hydroxybenzoic acid **31** (0.014), salicylic acid **32** (0.004), caffeic acid **35** (0.003), *o*-coumaric acid **36** (0.042), *p*-coumaric acid **37** (0.002), and ferulic acid **38** (0.007). The hydroxybenzoic and hydroxycinnamic acid compositions in this extract were much more than those found in the methanolic extract of flowers collected in Tunisia. Interestingly, quercetin **3** and naringenin **42** were commonly found in both extracts. However, this aqueous extract showed a weaker antioxidant activity than the ethyl acetate extract. This is perhaps due to the bulk content of non-active compounds, such as benzoic acid **24** (0.162) and caffeine **46** (0.1), thereby contributing to the high value of IC_50_.

Elansary et al. [13] screened their methanolic leaf extract in a DPPH assay, which resulted in the reported IC_50_ value of 17 μg/mL (Table 2). The HPLC-based analysis of their leaf extract revealed a high content of phytochemicals (reported in % *w*/*w* of crude extract). These were gallic acid **25** (0.136) and *p*-coumaric acid **37** (0.035). Other flavonoids such as rutin **6** (1.533), hyperoside **9** (0.633), isoquercetin **8** (0.073), quercetin **3** (0.006), and miquelianin **7** (0.126) were also identified. Isolated compounds such as quercetin **3**, rutin **6**, miquelianin **7**, isoquercetin **8**, hyperoside **9**, apigetrin **17**, gallic acid **25**, and *p*-coumaric acid **37** were also tested alongside the crude extract. The results indicated that high-content compounds such as rutin **6**, hyperoside **9**, miquelianin **7**, and gallic acid **25** exerted potent antioxidant activities, with IC_50_ values of 15, 4, 3, and 4 μg/mL, respectively. Evidently, these compounds were responsible for the high antioxidant activity exerted by the methanolic leaf extract. 

The work by Salem et al. [18] with a bark extract was shown to have potent antioxidant activity in a DPPH assay, which yielded an IC_50_ of 10.2 μg/mL compared to the positive control of ascorbic acid (IC_50_ = 7.66 μg/mL) (Table 2). Notably, the crude extract contained a higher content of phenolic acids and hydroxycinnamic acids than flavonoids. An HPLC-based analysis of the extract revealed (reported in % *w*/*w* of crude extract) gallic acid **25** (0.0255), benzoic acid **24** (0.0255), caffeine **46** (0.0106), and chlorogenic acid **40** (0.0106), followed by vanillin **29** (0.007), caffeic acid **35** (0.0054), rosmarinic acid **39** (0.005), and ferulic acid **38** (0.0042) as the main compounds. Flavonoids such as quercetin **3** (0.0037), kaempferol **22** (0.0011), and rutin **6** (0.0016) were also detected. 

Four different extracts from individual parts (flowers, leaves, and bark) of Australian *A. saligna* were obtained by our group using a sequential polarity-based extraction (Table 2). Bioactive extracts were determined using in vitro antioxidant DPPH assays [26]. Methanolic extracts from barks, leaves, and flowers were the most active. Compared to vitamin C (IC_50_ = 49.97 µg/mL), the bark extract was the most active, with an IC_50_ of 94.24 µg/mL, followed by the leaf (IC_50_ = 190.1 µg/mL) and then the flower (IC_50_ = 331.5 µg/mL). Pure compound isolation of the flower extract by column chromatography provided (reported in % *w*/*w* of dried flowers) naringenin **42** (0.183)**,** quercitrin **4** (0.432), naringenin-7-O-*α*-*L*-arabinofuranoside **47** (0.27), *D*-(+)-pinitol **48** (0.262), and isosalipurposide **1** (0.159). The leaf extract yielded (reported in % *w*/*w* of dried leaves) (–)-epicatechin **51** (0.0.91), quercitrin **4** (0.29), myricitrin **11** (0.507), 2,4-di-*t*-butylphenol **50** (0.101), (−)-pinitol **49** (0.913), and (3*S*,5*S*)-3-hydroxy-5-(2-aminoethyl)-dihydrofuran-2(3H)-one **52** (0.507). Isolation of the bark extract using column chromatography (reported in % *w*/*w* of dried bark) yielded (−)-epicatechin **51** (0.185), *D-*(+)-pinitol **48** (1.303), and sucrose (6.87).

The isolated compounds from each active extract were screened in the same DPPH assay. Myricitrin **11** (IC_50_ = 199.9 µM), (–)-epicatechin **51** (IC_50_ = 278 µM), and quercitrin **4** (IC_50_ = 322.6 µM) were the three active compounds among the isolated compounds. In similar DPPH methods, myricitrin **11** was reported to be active, with IC_50_ values ranging from 2.8 to 165.75 µM [40,41]. The (–)-epicatechin **51** was active with IC_50_ values ranging from 10.8 to 103.4 µM [42,43]. Similarly, the works by Li et al. [44] and Hong et al. [45] showed that quercitrin **4** had IC_50_ values of 4.45 and 107.5 µM. Naringenin **42**, naringenin-7-O-*α*-*L*-arabinofuranose **47**, and 2,4-di-*t*-butylphenol **50** showed poor activity against the DPPH, with undetectable IC_50_ values at the highest tested concentration of 10 mM. Similarly, this result was also observed for compound **52**. Naringenin **42**, as well as compounds **47**, **49**, **50,** and **52** performed poorly due to their lack of π-π conjugation OH groups required to donate hydrogen or electrons, which may have led to the possibility of forming radical resonance intermediates [46,47]. Naringenin **42** was also found to have poor activity by Cai et al. [46], where it yielded an IC_50_ of 2 mM. Isosalipurposide **1** was reported to be moderately active, with an IC_50_ of 81.9 µM in the DPPH assay [11]. These findings reiterate that IC_50_ values were considerably variable.

The presence of quercitrin **4** and perhaps naringenin **42** and isosalipurposide **1** (1.52% *w*/*w*) in the flower extract was responsible for the activity observed in this extract. The presence of three active antioxidants, namely, (–)-epicatechin **51**, quercitrin **4**, and myricitrin **11,** was reported to be supportive of the activity of the leaf extract. Quercitrin **4** and myricitrin **11** were also found in the leaf extract of Egyptian *A. saligna* [25]. The potent antioxidant activity of the leaf extract reported by Elansary et al. [13] was extensively supported by many other flavonoids and polyphenols in the extract, as indicated in their HPLC analysis. 

**Figure 5:**
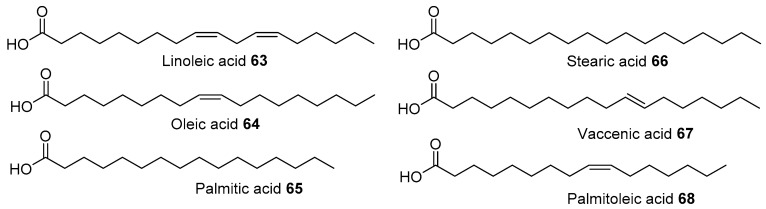
Fatty acids content of *A. saligna* seeds collected in Tunisia as determined via GC-MS analysis [48].

Youzbachi et al. [48] should also be mentioned for their investigation into the antioxidant of *A. saligna* seeds collected in Tunisia. Their methanolic extract displayed scavenging capacity in the DPPH assay, with an average IC_50_ value of 590 μM of Trolox equivalent antioxidant capacity (TEAC). The GC analysis of the methanolic extract revealed a high content of fatty acids, especially health-promoting fatty acids [reported in % of the total content (101.7 g/kg of dried weight (DW) of seeds], which included linoleic acid **63** (61.11–65.45), and oleic acid **64** (19.67–22.85). Smaller proportions of palmitic **65** (9.18–9.98), stearic **66** (1.49–1.82), vaccenic **67** (1.13–2.05), and palmitoleic **68** (0.34–0.58) acids were also quantified (Figure 5). The phenolic content of the extract was reported to be 1.91 g of gallic acid **25** equivalent kg^−1^ DW, while the total flavonoid content was shown to be in an average of 0.40 g of rutin **6** equivalent kg^−1^ DW. In addition, the group used the Kjeldahl assay to estimate the protein content of the seeds. The assay revealed a high content of protein, with an average of 107.2 g kg^−1^ on DW of seeds. This study did not conduct a detailed chemical analysis of the phenols and flavonoids to allow for further discussion. However, the estimated phenolic and flavonoid contents were evidently linked to the scavenging capacity observed in the seed extract. Notably, markedly high contents of linoleic acid **63** (61.11–65.45%) and oleic acid **64** (19.67–22.85%) were identified in the seed extract. These unsaturated fatty acids are known to benefit cardiovascular health [49].

The antioxidant activities (IC_50_ values) listed in Table 2 are markedly variable, even from the same plant part. For instance, the extracts of flowers collected in different parts of the world, when applying different extraction conditions, have been shown to have different IC_50_ values. The main contributor to this variability would be the different chemical compositions in each extract, as shown in Table 2 and the Supplementary Table S1. Although one can observe a few common compounds existing across the extracts, the compositions are variable. This information reiterates that factors, including location, growing conditions, collection season, and extraction methods, could influence the composition of metabolites in the extracts and, hence, the outcome of activities. 

Notably, three main groups of compounds were commonly identified (with variable concentrations) across actives extracts listed in Table 2. These are (i) flavonoids—isosalipurposide **1,** quercetin **3**, kaempferol **22**, rutin **6**, and naringenin **42;** (ii) hydroxycinnamic acids—caffeic acid **35**, *o*-coumaric acid **36**, *p*-coumaric acid **37**, and ferulic acid **38**; and (iii) benzoic acid **24** and hydroxybenzoic acids such as gallic acid **25,** salicylic acid **32,** ellagic acid **44,** syringic acid **28**, and *p*-hydroxybenzoic acid **31**. 

#### 3.1.1. Antioxidant Mechanism of Identified Compounds in *A. saligna*

**Scheme 1:**
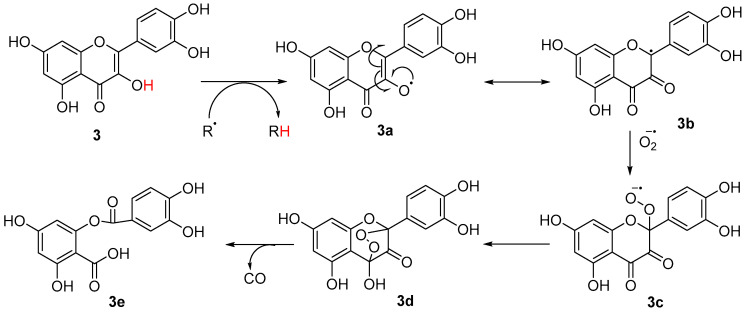
Possible antioxidant mechanism of quercetin **3** with a radical species (R^●^ and O_2_^−●^) [53,54].

The structural features of the flavonoids and phenolic acids required for antioxidant activity have been extensively investigated [39,46,47,50,51,52]. An antioxidant compound must donate hydrogen or a single electron to a reactive free radical to form an inactive species. When forming a resonance-stabilised radical intermediate that is comparatively more stable than the free radical, it becomes reduced. The overall result is the termination of damaging oxidative chain reactions. An example of the antioxidant mechanism of how flavonoids such as quercetin **3** can terminate the radical chain reaction is shown in Scheme 1 [53,54]. Quercetin **3** can donate a proton and an electron to a free radical (R^●^) from its phenolic (OH) groups to form resonance stabilised **3a**–**3b**. The intermediate **3b** then reacts with a radical (O_2_^−●^) to form **3c**, which can undergo ring epoxidation to provide **3d**. The decarboxylation of **3d** results in the formation of the stable benzoic derivative **3e**. The overall process leads to the termination of the damaging radical chain reactions.

#### 3.1.2. SAR of Antioxidants Compounds Identified in *A. saligna*

**Figure 6:**
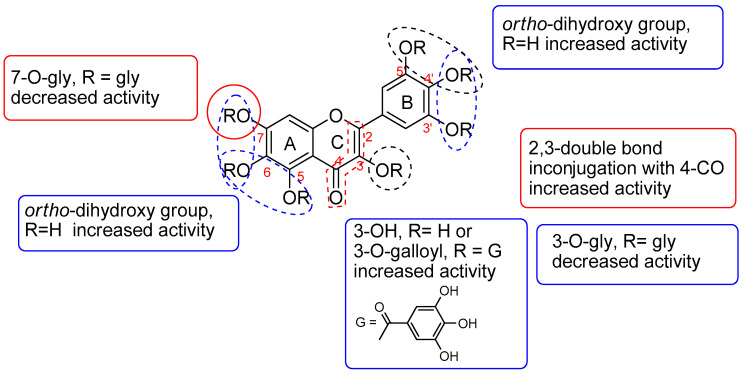
Structure–activity relationship of flavonoids required for antioxidant activity.

For flavonoids, the structure features required for antioxidant activity depend on the number and positions of hydroxyl (OH) groups, as well as other substituents and the glycosylation of the flavonoid nucleus [39]. Three structural features are essential determinants of the radical-scavenging activity of flavonoids: (i) the *ortho*-dihydroxy structure in the B-ring, (ii) the C2=C3 double bond in conjugation through the flavonoid nucleus, and (ii) the 4-oxo function in the C-ring. Flavonoids form complexes with the metal ions using the 3- or 5-hydroxyl and 4-keto substituents or hydroxyl groups in the *ortho* position in the B-ring, thus resulting in improved antioxidant efficacy. Studies of rare earth metal-chelated flavonol complexes confirmed that, in stable complex structures, the metal cations were found to chelate to 3-hydroxy and 4-keto sites [55,56,57]. Flavone glycosylation of the flavonoids at the 3- or 7-position diminishes their activity compared to the corresponding aglycones [39,52]. A systematic analysis of the SAR of flavonoid requirements for antioxidant activity is shown in Figure 6.

Hydroxycinnamic acids were shown to have in vitro antioxidant activities against human low-density lipid (LDL) oxidation. The activities of these compounds were ranked as caffeic acid **35** > ferulic acid **38** > *p*-coumaric acid **37**. The presence of the *ortho*-dihydroxy group in the phenolic ring (as in caffeic acid **35**) was suggested for the antioxidant activity of hydroxycinnamic acids toward LDL in vitro oxidation [58]. The radical scavenging activity of these hydroxycinnamic acids follows a similar mechanism to the flavonoids based on their ability to donate hydroxyl hydrogen and/or an electron to form resonance-stabilised intermediates. The scavenging mechanism is supported by (i) the *ortho*-dihydroxy phenyl configuration, which induces a metal ion chelation similar to that of flavonoids, (ii) the presence of –CH=CH–COOH groups in hydroxycinnamic acids that enhance the H-donating ability, and subsequent radical stabilisation due to the resonance-stabilised structure of α-β-unsaturated carbonyl [50,59].

For phenolic acids (hydroxybenzoic acid), the radical scavenging activity of the phenolic acids depends on the number and position of the hydroxyl (OH) groups and methoxy (–OCH_3_) substituents on the aromatic ring. The presence of π-π conjugation and *para*- and *ortho*-OH or –OCH_3_ increases the possibility of forming resonance-stabilised intermediates after proton abstraction or electron donation. Benzoic acid shows no radical scavenging capacity (0 mM TEAC) because of its absence of a hydroxy group and the inability to form resonance-stabilised structures in comparison to its hydroxyl derivatives, such as gallic acid **25**, methyl gallate **26**, syringic acid **28**, vanillin **29**, protocatechuic acid **30**, *p*-hydroxybenzoic **31,** and salicylic acid **32**, which have been identified across the antioxidant extracts of *A. saligna*. These hydroxybenzoic acids have been known to have substantial antioxidant activities. For instance, gallic acid **25** (3,4,5-trihydroxy-benzoic acid) was reported to have the most potent radical scavenging capacity (3.52 mM TEAC) because of the 3,4,5-trihydroxy configuration advantage and potent H-donating ability compared to protocatechuic acid **30** (IC_50_ = 1.15 mM), syringic **28** (4-OH, 3,5-OCH_3_, 1.39 mM), vanillic acid **29** (4-OH, 3-OCH_3_, 0.092 mM), and *p*-hydroxybenzoic acid **31** (0.03–0.04 mM) [52]. The presence of these compounds, therefore, provided a sufficient phytochemical link regarding the antioxidant activities observed in the extracts from *A. saligna*. 

In summary, the presence of flavonoids, hydroxycinnamic acids, and hydroxybenzoic acids identified across the extracts (Table 2) from various parts of the plant has provided ample support for the antioxidant capacity of these extracts.

### 3.2. Antibacterial

Several authors evaluated the antibacterial activities of extracts from *A. saligna*. The aqueous extract of flowers was shown to be active against phytopathogenic bacteria by Al-Huqail et al. [12]. The extract was active against *Agrobacterium tumefaciens* (MIC = 200 µg/mL), *Enterobacter cloacae* (MIC = 300 µg/mL), *Erwinia amylovora* (MIC = 300 µg/mL), and *Pectobacterium carotovorum* subsp (MIC = 100 µg/mL) (Table 3). The minimum inhibition concentration (MIC) values were lower than the positive control (Tobramycin in 10 µg/disc). HPLC-based analysis of the extract revealed two main phenolic and flavonoid compounds (Table 3). Among these (reported in % *w*/*w* of crude extract) were benzoic acid **24** (0.162), *p*-hydroxybenzoic acid **31** (0.014), syringic acid (0.006), and salicylic acid **32** (0.004). Hydroxycinnamic acids such as *o*-coumaric acid **36** (0.042), ferulic acid **38** (0.007), caffeic acid **35** (0.003), *p*-coumaric acid **37** (0.002), ellagic acid **44** (12.17), and catechol **45** (6.54) were also identified. The identified compounds were not isolated to confirm their antibacterial activity in pure form. Different work by the same group showed that pure caffeic acid **35**, *p*-coumaric acid **37**, and ferulic acid **38** inhibited the growth of *P. carotovorum* subsp at concentrations ranges of 800–3200 μg/mL [60]. 

Benzoic acid **24**, *p*-hydroxybenzoic acid **31**, and salicylic acid **32** were reported to completely inhibit *E. coli* lpxC/tolC strains at a concentration of 1 mg/mL. Furthermore, these benzoic acids were active again in the Gram-positive bacteria *S. aureus* EP167 strain with 100% inhibition at 1 mg/mL [61]. Benzoic acid **24** and its hydroxyl derivatives were active against Gram-negative bacteria due to their ability to cross the bacteria’s hydrophilic outer membrane and exert their antibacterial properties [62].

Hydroxycinnamic acids such as caffeic acid **35**, *o*-coumaric acid **36**, *p*-coumaric acid **37,** and ferulic acid **38** were reported to have broad-spectrum antibacterial activities against Gram-positive and Gram-negative pathogen bacteria, including the *Mycobacterium tuberculosis* (H37Rv) [63]. For instance, *o*-coumaric acid **36**, *p*-coumaric acid **37**, caffeic acid **35**, and ferulic acid **38** were shown to be active against the *E. coli* 0157:H7 strain with MIC values of 2.74 μM, 2.74 μM, 1.94 mM, and 2.23 mM, respectively [64]. *o*-Coumaric acid **36** and *p*-coumaric acid **37** were tested against *M. tuberculosis* H37Rv and showed MIC values of 122 μM and 244 μM, respectively [65]. *o*-Coumaric acid **36**, *p*-coumaric acid **37,** and caffeic acid **35** were shown to be active against *S. aureus* #917 with MIC values of 760 μM, 761 μM, and 694 μM, respectively [66].

Ferulic acid **38** was also reported to be active against the pathogens of concern, such as *Enterococcus faecalis* ATCC 2921 (MIC = 659 uM), *Klebsiella pneumoniae* RSKK 574 (MIC = 1.3 mM) [67], and *Pseudomonas aeruginosa* ATCC 10,145 (MIC = 515 μM) [68]. The literature indicates that hydroxycinnamic acids displayed marked antibacterial properties. A possible mechanism for their inhibition of bacterial growth or bactericidal properties is their damage to the bacteria’s cell wall [69]. Ferulic acid **38** and *p*-coumaric acid **37** were shown to interact with the cell membranes of the bacteria and disrupt the phospholipid or lipid bilayers. The loss of cell membrane integrity resulted in the formation of pores and increasing membrane permeability [70]. In addition, *p*-coumaric acid **37** was shown to disrupt membrane permeability and create pores in *E. coli*, *S. dysenteriae*, and *S. Typhimurium* [71]. These antibacterial compounds, therefore, sufficiently support the antibacterial activities exerted by the crude extracts of *A. saligna*.

Elansary et al. [13] were the first to determine which phytochemicals contributed to the antibacterial activities observed in their methanolic leaf extract (Table 3 and Table 4). The extract was shown to inhibit the growth of *B. cereus, P. aeruginosa, Listeria monocytogenes*, *E. coli*, *Micrococcus flavus*, and *S. aureus* with MIC values of 0.35, 0.37, 0.47, 0.31, 0.41 and 0.30 mg/mL, respectively, compared to the positive control streptomycin (MIC = 0.07–0.15 mg/mL). The extract was also bactericidal against all the tested bacteria, with minimum bactericidal concentration (MBC) values of 0.73, 0.79, 0.99, 0.72, 0.85, and 0.73 mg/mL, respectively (Table 4). HPLC-based analysis of their leaf extract revealed a high content of phytochemicals (reported in % *w*/*w* of crude extract, as shown in Supplementary Table S1), which revealed rutin **6** as the main flavonoid in the extract. Pure rutin **6**, hyperoside **9**, *p*-coumaric acid **37**, quercetin **3**, and miquelianin **7** were screened against the tested bacteria alongside the crude methanolic extract, as shown in Table 4. Rutin **6** and *p*-coumaric acid **37** showed substantial broad-spectrum antibacterial activities against tested bacteria, which were comparable to the positive control streptomycin. In contrast, hyperoside **9**, quercetin **3**, and miquelianin **7** showed moderate to low antibacterial activities. The antibacterial activities of compounds, especially rutin **6** and *p*-coumaric acid **37**, supported the activity observed in the crude extract. In addition, the flavonoids such as rutin **6**, quercetin **3**, miquelianin **7**, and isoquercetin **8** have been reported to have antibacterial properties [72]. As previously discussed, gallic acid **25** and *p*-coumaric acid **37** are known antibacterial compounds.

El-Toumy et al. [16] reported the antibacterial activities of their EtOAc leaf extract against Gram-positive bacteria, including *S. aureus*, *Streptococcus pyogens*, *B. cereus*, and *B. subtilius* with MIC values of 0.41, 0.46, 0.41, and 0.14 μg/mL, respectively. Interestingly, the ethyl acetate leaf extract was much more active against *S. aureus* and *B. cereus* than the methanolic leaf extract reported by Elansary et al. (Table 4) [13]. 

The leaf extract from the El-Toumy group was subjected to pure compound isolation by column chromatography to yield sixteen polyphenolic compounds. The compounds were structurally elucidated by NMR spectral data analysis to mainly provide flavonoids [16]. Among these were (reported in % *w*/*w* of crude extract) quercetin **3** (0.025), quercitrin **4** (0.030), quercetin-3-*O*-arabinoside **5** (0.030), myricetin **10** (0.025), myricitrin **11** (0.034), myricetin-3-*O*-arabinoside **12** (0.028), myricetin-3-*O*-glucopyranoside **13** (0.029), (+)-catechin **14** (0.025), 7-*O*-Galloyl-cathecin **15** (0.029), apigenin **16** (0.002), apigetrin **17** (0.002), luteolin **19** (0.029), cynaroside **20** (0.027), gallic acid **25** (0.027), and methyl gallate **26** (0.030), as shown in Table 3 and Supplementary Table S1. 

The work by Gumgumjee et al. [17] on the ethanolic extract of the leaves collected in Saudi Arabia was active against resistant Gram-positive bacteria such as *Micrococcus* and *Methicillin-resistant S. aureus* (MRSA) at 200 mg/mL. The most susceptible bacteria were *K. pneumonia*, MRSA, and *B. subtilius*. The diameters of the inhibition zones of the extract against these bacterial strains were 29.33 mm, 27.66 mm, 22.66 mm, and 23.33 mm, respectively. The positive control streptomycin inhibition zones against the above bacteria were 25 mm, 20 mm, 19 mm, and 27 mm, respectively. The results indicated the extract was as active as the 10 μg/disc of streptomycin. The extract was also reported to be active against the pathogenic Gram-negative *E. coli* and *P. aeruginosa* with inhibition zones of 25.66 mm and 25.66 mm, respectively. The inhibition zones were compared to the positive control 10 μg/disc of streptomycin, which suggested that the activity of the extract against these three Gram-negative bacteria was comparable to streptomycin. 

HPLC-based analysis of the active extract showed that it mainly contained hydroxybenzoic acids (reported in % *w*/*w* of crude extract) such as gallic acid **25** (0.00543), syringic acid **28** (0.00037), chlorogenic **40** (trace), *p*-hydroxybenzoic **31** (0.0002), vanillic acid **29** (0.0002), *p*-coumaric acid **37** (0.00083), and salicylic acid **32** (0.00013). The concentration of gallic acid **25** was the highest, followed by *p*-coumaric acid **37**, syringic **28**, and vanillic acid **29.** Similar to the works described earlier, these identified compounds are known to have significant broad-spectrum antibacterial activities [63,73] and were, therefore, responsible for the antibacterial activity observed in the crude extracts.

#### 3.2.1. Possible Mechanisms of Action (MOA) of Antibacterial Compounds Identified in *A. saligna*

Flavonoids’ MOAs for the inhibition or killing of bacteria has been suggested in three modes based on their structures [74]. The MOA of identified antibacterial compounds in *A. saligna,* such as quercetin **3**, kaempferol **22**, myricetin **10**, and rutin **6**, might occur via the destruction of the bacterial cytoplasmic membrane [75] by a perforation mechanism [76], decreasing membrane fluidity [77], or disrupting lipid bilayers and membrane barriers. They could also inhibit energy metabolism [78] by inhibiting NADH cytochrome c reductase. They might also inhibit nucleic acid synthesis [79] by inhibiting the DNA gyrase enzyme. 

Other suggested MOAs associated with bacterial resistance include inhibiting biofilm formation and e-flux pumps. For instance, rutin **6** at a 50 μg/mL concentration inhibited the biofilm formation of *E. coli, S. aureus* [80], and *Streptococcus suis* at 19.5 μg/mL [81].

#### 3.2.2. SAR of Antibacterial Flavonoids Identified in *A. saligna*

**Figure 7:**
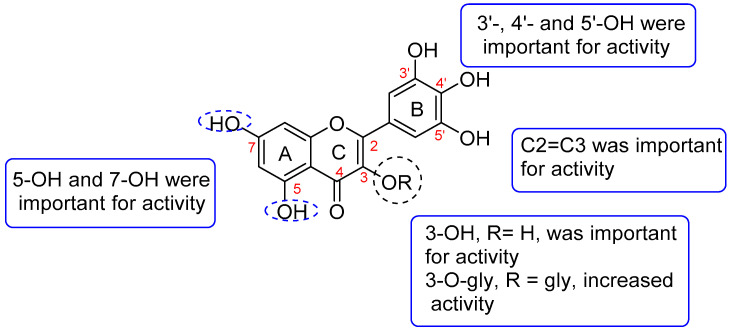
SAR of flavonoids required for antibacterial activities (adapted from Farhadi et al. [82] and Shamsudin et al. [83].

**Figure 8:**
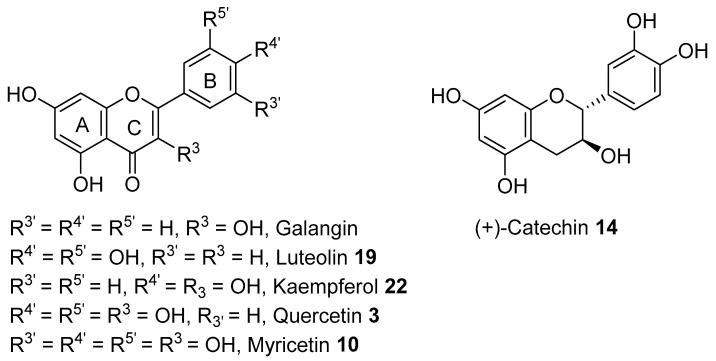
Structures of antibacterial flavonoids.

Comprehensive reviews by Farhadi et al. [82], Shamsudin et al. [83], and studies reported within the reviews revealed that different flavonoid structural configurations could exhibit different antibacterial activities. The SAR suggested that ring A and hydroxy groups at positions C-5 and C-7 were required for antibacterial activity against *S*. *aureus* strains (Figure 7) [84]. The presence of hydroxylation (number and position) on the B ring could influence the antimicrobial activities of these compounds, such as quercetin **3** (with 3′,4′-dihydroxy), kaempferol **22** (with 4′-hydroxy), and galangin (without a hydroxy at C-4′), as shown in Figure 8. Kaempferol **22** was found to be the most active inhibitor of *E. coli* DNA gyrase (MIC = 25 μg/mL), followed by quercetin **3** (MIC = 36 μg/mL), and the least active was galangin (MIC = 53 μg/mL), which demonstrated that 3′-OH decreased activity and emphasised the importance of 4′-OH in the ring B [77]. However, kaempferol **22** (4′-OH) was less active against Gram-positive bacteria than galangin [85].

Xu and Lee [86] investigated 38 plant-derived flavonoids against antibiotic-resistant bacteria such as MRSA, multidrug-resistant *Burkholderia cepacian,* and vancomycin-resistant enterococci (VRE); they showed that myricetin **10**, kaempferol **22**, quercetin **3**, and luteolin **19** were active against MRSA. In contrast, only myricetin **10** was found to be active on multidrug-resistant *B. cepacian* and VRE with MIC values of 32 and 128 μg/mL, respectively. The results highlight the importance of 3′,4′, and 5′-OH of ring B against multidrug-resistant bacteria. Furthermore, it was suggested that the C2=C3 double bond could also be necessary for the activity when the activity of quercetin **3** was compared with that of (+)-catechin **14**.

The 3-OH in ring C is considered essential for the activity; for instance, quercetin **3** (MIC_50_ = 36 μg/mL) was found to be more active against *E. coli* (DNA gyrase inhibition) than luteolin **19** (MIC_50_ 67 μg/mL), which lacks the 3-OH [77]. In addition, the presence of glycosylic at C-3 also significantly improved antibacterial activities compared to the 3-OH counterpart [87]. 

The antibacterial properties of the identified compounds in Table 3 and their discussed MOA and SAR explain and provide sufficient support for the antibacterial activities observed in the crude extracts of *A. saligna*.

### 3.3. Antifungal 

In addition to antioxidant and antibacterial properties, the aqueous extract from flowers of *A. saligna* investigated by Al-Huqail et al. [12] was also shown to have antifungal activities against three phytopathogen fungi, *Fusarium culmorum*, *Rhizoctonia solani*, and *Penicillium chrysogenum*, that are harmful to agriculture (Table 3). At 3% of the extract applied onto the *Melia azedarach* wood sample, it showed the inhibition of the mycelial growth of *F. culmorum*, *P. chrysogenum*, and *R. solani*, with values of 38.51%, 65.92%, and 41.48%, respectively, compared to the control (10% DMSO). As previously discussed, the HPLC-based analysis of the aqueous extract revealed that benzoic acid **24**, caffeine **46**, *o*-coumaric acid **36,** quercetin **3**, naringenin **42**, and kaempferol **22** were the most abundant.

Although screening of these pure identified compounds was not carried out, these identified compounds were reported to have antifungal activities. For instance, benzoic acid **24** and its hydroxyl derivatives are known to protect tomato plants from early blight disease caused by *Alternaria solani* [51,88]. Benzoic acid **24** and *p*-hydroxybenzoic acid **31** effectively inhibited the mycelial growth of *A. solani* with IC_50_ values of 44.69 and 58.80 ppm, respectively. Furthermore, benzoic acid **24** has been used as a preservative against fungi that cause food spoilage. Furthermore, benzoic acid **24** and its hydroxy derivatives have been known to have broad antifungal activity against soil borne fungi, such as *Fusarium* sp. [89], and seed-borne pathogens, such as *Aspergillus flavus*, *Penicillium citrinum*, and *Alternaria alternata* [90].

Therefore, benzoic acid **24** and its derivatives in the extract support the observed antifungal activities. The high concentration of benzoic acid **24** indicated the plant’s defence mechanism against fungal infection. Interestingly, benzoic acid **24** was also reported to be produced by the soil bacteria *Bacillus licheniformis* MH48 as a defensive second metabolite against pathogenic fungal infections, with a minimum inhibitory concentration of 128 mg/mL [88]. The identified flavonoids, such as naringenin **42** [91], quercetin **3,** and kaempferol **22** [92,93], are also known natural antifungal compounds. Therefore, their presence would synergistically support the fugal activities observed in this aqueous flower extract. 

Gumgumjee et al. [17] investigated the inhibitory activities of their EtOH extract of leaves collected in Saudi Arabia on the growth of fungi known to cause human infections; these were *Aspergillus niger, A. fumigatus*, *A. flavus* and *C. albican* (Table 3). The study was investigated using the disc diffusion method against the four fungi species at 200 mg/mL concentration compared to the positive controls amphotericin and nystatin (10 mg/disc). In decreasing order, the extract was active against the fungi; *A. fumigatus> C. albicans> A. flavus> A. niger.* The diameters of the inhibition zones of the extract against these fungi strains were 25.67, 23.33, 21.33, and 20.00 mm, respectively, compared to the average inhibition zones of amphotericin and nystatin of 27.00 and 29.75 mm, respectively. The results indicated that the antifungal activities of the extract at 200 mg/mL were slightly less than amphotericin at 10 mg. 

Elansary et al. [13] also screened their methanolic extract of leaves collected in Saudi Arabia against common pathogenic fungi such as *A. ochraceus*, *A. niger*, *A. flavus*, *C. albicans*, *P. ochrochloron*, and *P. funiculosum* with antifungal ketoconazole as the positive control (Table 5). The extract was active against the fungi in decreasing order: *A. flavus> A. ochraceus > P. funiculosum > P. ochrochloron > A. niger> C. albicans.* MIC values of the extract against these fungi strains were 0.30, 0.38, 0.43, 0.44, 0.48, and 0.58 mg/mL, respectively (Table 5). The extract was most active against *A. flavus* with an MIC value of 0.33 mg/mL compared to the positive control ketoconazole (MIC of 0.20 mg/mL). 

The leaf extract was also fungicidal across the tested fungi, with MFC values ranging from 0.91 to 1.42 mg/mL (Table 5). The identified pure compounds, such as rutin **6**, hyperoside **9**, miquelianin**7**, and *p*-coumaric acid **37,** were also screened. These compounds showed potent antifungal activities against all tested fungi, which were comparable to the positive control ketoconazole (MIC 0.10–2.05 mg/mL). As previously mentioned, rutin **6**, hyperoside **9**, gallic acid **25**, miquelianin **7**, isoquercetin **8**, *p*-coumaric **37**, and quercetin **3** were the main compounds detected in the HPLC-based analysis of the extract [13]. However, gallic acid **25** and isoquercetin **8** were not screened in the assay. Nevertheless, gallic acid **25** was reported to inhibit the growth of *A. solani* [51,88] and has been suggested as a green fungicide [94]. 

In addition, these identified compounds have been shown to have antifungal activities elsewhere against various phytopathogen fungi. For instance, rutin **6** and quercetin **3** were active against *C. albicans* [72,95]. Hyperoside **9** has been reported to be active against *Alternaria alternata*, *Pestalotia guepini*i, *Fusarium avenaceum*, *Drechslera* sp., and *Epicoccum nigrum* [96]. *p*-Coumaric acid **37** was reported to be active against *Botrytis cinnerrea* [97]. These phytochemicals identified by Elansary et al. [13] in their methanolic leaf extract (Table 3) would be synergistically responsible for the observed fungal activity.

The antifungal activities found in the methanolic leaf extract by Elansary et al. [13] were slightly different concerning the activity order compared to the ethanolic leaf extract investigated by Gumgumjee et al. [17]. Interestingly, the leaves used in both studies were collected in the same region (Saudi Arabia). However, the compositions of the identified compounds in the alcoholic extracts were markedly different (Table 3 and Supplementary Table S1). For instance, Gumgumjee et al. [17] mainly reported hydroxybenzoic acids and hydroxycinnamic acids in their extract. In contrast, the identified compounds in the extract of the Elansary group [13] were mainly flavonoids. The two common compounds between these leaf extracts were gallic acid **25** and *p*-coumaric acid **37**. Although these results cannot be directly compared, it is interesting to note that there is a similar trend in order of activity against *A. flavus* and *A. niger*.

The ethanolic extract of the bark collected in Egypt was reported by Salem et al. [18] to have fungicidal properties against six *Fusarium oxysporum* strains isolated from different plant hosts (Table 3). The MIC values observed across the six *F. oxysporum* were 64–125 μg/mL, compared to the activity of the reference fungicide carbendazim (MIC 5–10 μg/mL). HPLC analysis of this bark extract revealed the presence of (reported in % *w*/*w* of crude extract) gallic acid **25** (0.0255) and benzoic acid **24** (0.0158) as the two major compounds. Other identified compounds were caffeine **46** (0.0107), chlorogenic acid **40** (0.0104), vanillin **29** (0.0067), caffeic acid **35** (0.0054), rosmarinic acid **39** (0.0054), ferulic acid **38** (0.0042), quercetin **3** (0.0037), rutin **6** (0.0016) *o*-coumaric acid **36** (0.0011), kaempferol **22** (0.001), and *p*-coumaric acid **27** (0.0008). 

In summary, among the four antifungal extracts (Table 3), it is interesting to note that the dominant compounds were gallic acid **25** and benzoic acid **24.** Gallic acid **25** was found in the leaf, while benzoic acid **24** was only found in the flower extracts. As previously discussed, these two compounds are known antifungals. The three identified flavonoids, quercetin **3**, rutin **6**, and kaempferol **22**, were commonly found between the leaf and flower extracts (Table 3). It is essential to note that the wide variety of cinnamic acids also contributed to the observed antifungal activities of the extracts.

### 3.4. Inhibition of α-Glucosidase Enzyme

Although many research groups have reported the potent antioxidant properties of *A. saligna*, the plant’s antidiabetic property is under investigation. Diabetic disorders such as type 2 diabetes (T2D) are driven by metabolic abnormalities connected to obesity and impaired insulin response, together with the rise of hepatic glucose production, thus ultimately disrupting glucose homeostasis [98,99]. Previous studies have shown that compounds with antioxidant activity can improve glycaemic control in animal models of T2D [100]. Furthermore, one known therapy for T2D is the inhibition of key digestive enzymes related to diabetes, namely, human *α*-amylase and *α*-glucosidase and their inhibitors, such as acarbose, voglibose, and miglitol. These synthetic inhibitors have been known to have adverse side effects, namely, hepatotoxicity and gastrointestinal symptoms [101]. It would be beneficial to find safer and more affordable alternatives. Studies have revealed that the inhibition of *α*-glucosidase enzyme and antioxidant activity could be achieved using natural products such as flavonoids [102].

Buttner et al. [19] recently reported the yeast α-glucosidase and porcine pancreatic *α*-amylase inhibitory activities of their ethanolic bark and leaf extracts from *A. saligna* harvested in South Africa. The leaf extract showed inhibition against α-glucosidase and α-amylase with IC_50_ values of 2.35 µg/mL and 10.45 µg/mL, respectively. The IC_50_ of the bark extract against α-glucosidase was 3.64 µg/mL, which was relatively similar to that of the leaf extract. In comparison, the activity of the bark extract against *α*-amylase was weaker than that of the leaves (IC_50_ = 17.67 µg/mL). The IC_50_ values of all extracts were much lower than the positive control acarbose (IC_50_ = 330.71 µg/mL). Only Folin–Ciocâlteu reagent analysis was carried out on these active extracts, and they were shown to have high flavonoid and phenolic contents. As indicated in the earlier sections (Table 2 and Table 3), various compounds such as quercetin **3**, rutin **6**, miquelianin **7**, isoquercetin **8**, hyperoside **9**, gallic acid **25**, and *p*-coumaric acid **37** were identified in the crude leaf extracts of *A. saligna* harvested in Egypt [13]. HPLC-based analysis of the bark extract carried out by Salem et al. [18] revealed the presence of quercetin **3**, rutin **6**, kaempferol **22**, benzoic acid **24**, gallic acid **25**, vanillin **29**, caffeic acid **35**, *o*-coumaric acid **36**, *p*-coumaric acid **37**, ferulic acid **38**, rosmarinic acid **39**, chlorogenic acid **40**, and caffeine **46**. These compounds might also be present in the bark and leaf extracts investigated by Buttner et al. [19].

Recent work by our group reported that the methanolic extracts were obtained via a sequential polar-based extract from the flowers, leaves, and bark of *A. saligna* collected in Australia. The methanolic extracts were active against yeast α-glucosidase compared to their non-polar counterpart. The methanolic bark extract showed superior α-glucosidase inhibitory activity (IC_50_ 4.373 µg/mL) compared to acarbose (IC_50_ = 254 µg/mL) (Table 6) [26]. This value was comparable with the inhibitory activity of crude ethanolic bark and leaf extracts of the South African *A. saligna* [19]. 

Although the yeast enzyme was commonly used as an in vitro initial screening of medicinal plants for their α-glucosidase inhibitory activities due to the commercial availability of the enzyme, it is essential to note that an inhibitor, for instance, acarbose, was reported to exert more inhibitory activity against mammalian *α*-glucosidase enzymes than the yeast enzyme. Furthermore, Pacillia et al. [103] reported that naringenin **42** displayed an effective inhibition against the yeast enzyme (IC_50_ = 6.51 μM). However, when tested on the rat intestinal glucosidase, it was poorly active (IC_50_ = 384 μM). Therefore, the mammalian enzymes from rats or from humans should be considered when discovering candidates for human application.

The yeast α-glucosidase inhibitory activities of isolated compounds from the three active extracts were determined using the same procedure for the extracts. The IC_50_ values of isolated compounds are shown in Table 6. (–)-Epicatechin **51** (IC_50_ = 63.58 μM), *D*-(+)-pinitol **48** (IC_50_ = 74.69 μM), naringenin **42** (IC_50_ = 89.71 μM), isosalipurposide **1** (IC_50_ = 116.5 μM), (–)-pinitol **49** (IC_50_ = 164.2 ± 8.362 μM), and quercitrin **4** (IC_50_ = 177.3 ± 11.34 μM) inhibited the enzymes better than compounds **47**, **50**, **11**, and the positive control acarbose. Notably, *D*-(+)-pinitol **48** was a potent inhibitor that was two times more active than its enantiomer (–)-pinitol **49**. 

*D*-(+)-Pinitol **48** has been used as a natural health supplement to provide therapeutic benefits in treating T2D. It is also a natural antidiabetic agent and insulin regulator with anti-inflammatory [104] and hepatoprotective [105] activities. In an animal model of diabetes, *D*-(+)-pinitol **48** has been described as an antidiabetic agent with an insulin-like effect that can enhance insulin activity by translocating the GLUT-4 in the mice’s skeletal muscles [106,107]. It was demonstrated in animal and clinical studies that *D*-(+)-pinitol **48** effectively regulated hyperglycemia and prevented insulin resistance. The mechanism of action of *D*-(+)-pinitol **48** was explained via its ability [108] (i) to protect the pancreas against diabetes-induced oxidative stress because of its antioxidation property [104]; (ii) to overcome insulin resistance by modulating the PI3kt/Akt signalling pathway in a rat model [109]; and (iii) to increase glucose-induced insulin secretion by reducing the expression of the subunit alpha of AMP-activated protein kinase (AMPK-*α*) that protected against triglyceride deposition in the liver [110]. The inhibitory activity of *D*-(+)-pinitol **48** against α-glucosidase would provide an additional therapeutic benefit to combat T2D.

Studies have revealed that the inhibition of the *α*-glucosidase enzyme could be achieved using flavonoids [102]. Proença et al. [111] reported the *α*-glucosidase inhibitory activity of flavonoids, particularly those identified in *A. saligna*. Quercetin **3**, kaempferol **22**, and luteolin **16** were found to have IC_50_ values of 15, 32m and 46 µM, respectively, compared to the positive control acarbose (IC_50_ = 607 µM). At the same time, rutin **6** and naringenin **42** were found to inhibit the enzyme activity by <20% and 45% at 200 µM (the highest tested concentration). Another group showed that myricetin **10**, quercetin 3, kaempferol **22**, luteolin **16**, and naringin **42** inhibited the enzyme with IC_50_ values of 5, 7, 12, 21, and 75 µM, respectively [112]. Myricetin **10** was active with an IC_50_ of 4.48 µM compared to the positive control acarbose (304 µM) [113]. Kaempferol **22** has also been shown to inhibit the enzyme with an IC_50_ value of 77.42 μM [102].

#### SAR of Flavonoids Required for the α-Glucosidase Inhibition

**Figure 9:**
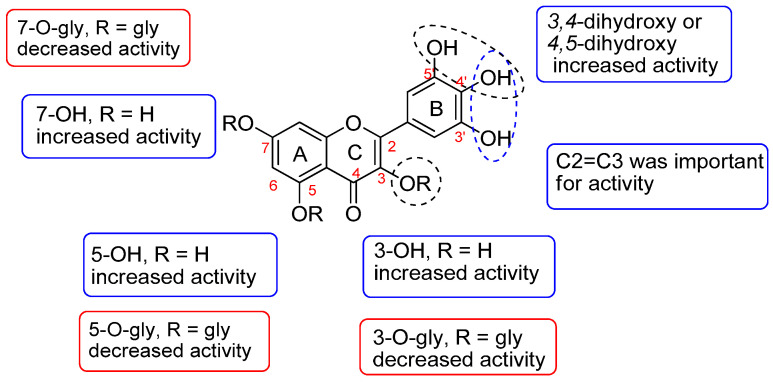
SAR of flavonoids required for *α*-glucosidase inhibition (adapted from Proença et al. [111] and Şöhretoğlu et al. [102]).

SAR investigation of flavonoids carried out by Proença et al. [111] suggested that flavonoids with two phenolic groups at the A or B ring and a hydroxy group at C-3 possessed the highest α-glucosidase inhibitory activity. He et al. [114] and Şöhretoğlu et al. [102] further reiterated that the number of phenolic groups on ring B was vital for the activity. A free hydroxyl group at C-4′ was crucial for *α*-glucosidase inhibition. An additional hydroxy group at C-3′ and C-5′ generally increased the effect (Figure 9). 

The double bond C2=C3 and a free hydroxyl group at C-4′ were crucial for *α*-glucosidase inhibition. The hydroxylation of flavonoids generally increased the effect, though the effects of an additional hydroxyl group at C-3′ were debatable. 

Adding a sugar moiety to aglycon usually reduced the inhibitory effect; however, further phenolic acid substitutions to these sugar units restored the inhibitory effect. Based on structure-based molecular modelling studies, it is possible to conclude that hydroxyl groups at C-5 and C-7 positions, the carbonyl oxygen at the C-4 position, and hydroxyl groups in ring B were among the suggested key groups to enhance binding to the enzyme through H-bonds. Their docking study indicated that the B ring of the flavonoids located deep inside the active side of the enzyme and the presence of the phenolics significantly improved interaction via hydrogen bonding. On the other hand, bulky flavonoid glycosides showed poor inhibition due to their inability to access the binding pocket.

### 3.5. Anti-Inflammation

Investigation of the *n*-butanol extract from the shoots of *A. saligna* collected in Egypt was investigated for its anti-inflammation potential in the rat model of acetic acid-induced ulcerative colitis (UC) [115]. The specific application is an alternative to corticosteroids and 5-aminosalicylic acid, which are commonly used to treat inflammatory bowel diseases (IBD) and UC. Nano-formulation of the *n*-butanolic extract as silver nanoparticles (Ag-NPs) was also investigated. The extract and nano-formulation form showed the protection of colon tissue from inflammation, ulceration, wall thickening, necrosis, and gangrenous changes in colon tissue. Treatment of the extract also reduced Cyclooxygenase-2 (COX-2), Prostaglandin E2 (PGE_2_), and Interleukin-1 (IL-1β) levels in the inflamed colon induced by acetic acid. The nano-formulation of the extract appeared to perform better than the *n*-butanolic extract in protecting tissue against inflammation and was comparable to the reference drug dexamethasone. The nano-extract restored metabolite (lactic acid, fructose, and pyroglutamic acid) levels to normal, thus suggesting that cytokine levels were regulated by the nano-extract in UC. 

Six flavonoids and nine phenolic compounds were identified in the extract. They were naringenin **42**, taxifolin **41**, (+)-catechin **14**, quercetin **3**, rutin **6**, kaempferol **22**, gallic acid **25**, methyl gallate **26**, syringic acid **28**, cinnamic acid **34**, ferulic acid **38**, coumaric acid **37**, caffeic acid **35**, ellagic acid **44**, and chlorogenic acid **40**. Although the compounds were not screened for their specific inhibitory activities, naringenin **42**, taxifolin **41**, (+)-catechin **14**, quercetin **3**, rutin **6**, and kaempferol **22** were known to have antioxidant and anti-inflammatory activities [116]; their therapeutic activities have been considered to be beneficial for the treatment of BID [117]. Mainly, naringenin **42**, quercetin **3**, and kaempferol **22** have been known to inhibit COX-2, IL-1β and nuclear factor kappa-B (NF-κB) activities [118,119,120]. Rutin **6** is capable of down-regulating COX-2 and NF-κB activities [121,122,123]. The literature has provided ample evidence to indicate that the identified flavonoids in the extract are responsible for inhibiting inflammatory biomarkers of PGE_2_, COX-2, and IL-1β in ulcerative colitis in a rat model induced by acetic acid [115]. The authors also suggested that loading bioactive extracts in nanoparticles was one of the strategies to enhance the extract’s efficacy via increasing the active compounds’ biostability and absorption in the tissues [124]. The anti-ulcer effect of the extract and its nano-formulation in the rat model highlighted the potential of *A. saligna* and its identified compounds as new therapeutics for acute colitis.

### 3.6. Anticancer

Elansary et al. [13] screened their methanolic leaf extracts and identified compounds (Table 5) against cancerous cell lines such as colon adenocarcinoma (HT-29), cervical adenocarcinoma (HeLa), breast adenocarcinoma cultures (MCF-7), and human T-cell lymphoblasts (Jurkat) in the 3-(4,5-dimethylthiazol-2-yl)-2,5-diphenyl-tetrazolium bromide (MTT) assay. The results indicated that the extract showed strong antiproliferative effects against the tested cell lines with the most potent activity against HeLa (IC_50_ = 33 μg/mL) > HT-29 (IC_50_ = 36 μg/mL) > MCF-7 (IC_50_ = 59 μg/mL) > Jurkat (IC_50_ = 62 μg/mL). The identified compounds also exhibited antiproliferative activities against the tested cell lines in the extract. For instance, rutin **6**, hyperoside **9**, quercetin **3**, miquelianin **7**, and *p*-coumaric acid **37** showed inhibition of HeLa with IC_50_ values 4, 7, 5, 5, and 7 μg/mL, respectively, compared to the positive control vinblastine sulphate (IC_50_ = 2 μg/mL). The author suggested that the observed cytotoxic effects in the crude extract was due to rutin **6**, hyperoside **9**, quercetin **3**, quercertin-3-glucuronide **7**, and *p*-coumaric acid **37**. Both rutin **6** and hyperoside **9** have been known to have antiproliferative and cytotoxic properties against cancer cells [72,125,126].

Gedara et al. [25] reported the cytotoxic activity of isolated compounds from the methanolic leaf extract against liver cancer cells (HEPG2) in a sulphorhodamine B staining method [127]. The study used adriamycin as a positive control (IC_50_ = 0.54 mg/mL). Spirostane saponin **55** inhibited cell growth with an IC_50_ of 2.8 μg/mL, while erythrodiol **53** (IC_50_ = 11 μg/mL) and **54** showed moderate activities (IC_50_ = 11.2 μg/mL) (Figure 3). On the other hand, flavonoid glycosides such as myricitrin **11**, quercitrin **4**, and biflavonoid **33** (Figure 2) exhibited weak cytotoxic activities with IC_50_ values of 14, 17.2, and 17.8 μg/mL, respectively.

## 4. Toxicity of Bioactive Extracts and Their Safety 

To apply the extracts in drug development, the crude extracts must be non-toxic towards non-cancerous mammalian cells. Elansary et al. [13] reported the non-toxic activity of their crude methanolic extract of *A. saligna* leaves from Saudi Arabia against HEK-293 (non-cancerous human embryonic kidney cells). Their MTT assays revealed no significant toxicity against HEK-293 cells at the highest tested concentration of 400 μg/mL. Buttner et al. [19] also reported the non-toxicity of their leaf and bark extracts of *A. saligna* from Saudi Arabia against human colorectal adenocarcinoma (Caco-2) cells at the highest tested concentration of 300 mg/mL, wherein they used the Hoechst 33,342 and propidium iodide dual staining method. Our recent work on methanolic extracts from flowers, leaves and bark collected in Australia revealed that these extracts showed no toxicity against 3T3-L1 adipocytes at 200 μg/mL after incubation for 72 h in an MTT assay [26]. The 3T3-L1 adipocytes were used in this study because they are the ideal cell model suitable for investigating the antidiabetic activities of *A. saligna.* The cell line provides an excellent model of white adipose tissue to investigate glucose uptake, lipogenesis, and glycogen synthesis under an insulin-resistant state [128]. These findings have highlighted the non-toxicity potential of *A. saligna*. 

This review has highlighted in vitro bioactivities of *A. saligna*’s extracts and isolated compounds. Although the non-toxicity of the plant extracts in cell-based has been discussed here, various critical issues such as drug safety, effectiveness, and suitable administration must be first addressed before the plant can be suitable for clinical applications. The process would involve preclinical and clinical trials to determine the toxicity, drug metabolism, formulation, and stability of the plant’s extracts. 

Nevertheless, some identified flavonoids in the extracts have been used as health supplements. The extract comprises a mixture of compounds, including those reported in the literature and others yet to be identified. If these phytochemicals can have synergetic effects against the targets, leading to therapeutic outcomes without side effects. In contrast, the interactions of compounds could also lead to adverse side effects. Hence their safety and effectiveness need to be cautiously investigated. At this point, we consider that the extracts from *A. saligna* are valuable and suitable for further investigation into investigations of its potential for treating inflammation, diabetes, and metabolic-related diseases.

## 5. Conclusions

Antioxidant, antimicrobial, anticancer, α-glucosidase inhibition, and anti-inflammatory activities of *A. saligna* have been summarised. Benzoic acids, cinnamic acids, and flavonoids were the most frequently documented active phytochemicals from *A. saligna* extracts. Other compounds, such as unsaturated fatty acids, saponins, and pinitols, were minor but equally had high therapeutic potential. The compositions and quantities of identified compounds varied depending on the plant parts, geographic origins (growing conditions), extraction solvents, and analytic methods. The differences in chemical compositions reflected the variability of biological activities exhibited by extracts, even in a similar assay. The biological activities of these identified compounds provided ample support for the activities exerted by the extracts.

The SAR of identified compounds revealed critical structural features required for their bioactivities. For instance, these include the structural features required of flavonoids for antioxidant activities depended on the number and positions of hydroxyl (OH) groups and other substituents and the glycosylation of the flavonoid nucleus. The hydroxyl groups were vital for the proton and electron transfer reaction in the scavenging of free radicals; the formation of non-covalent interactions in antibacterial, antifungal, and α-glucosidase inhibition; and the induction of anticancer and anti-inflammatory cell signalling pathways. The conjugated C=C of flavonoids, hydroxybenzoic, and cinnamic acids were essential for electron delocalisation and the formation of resonance-stabilised intermediates. These structural features could interact with key amino acid residues at the active site of *α*-glucosidase. Depending on the position, glycoside substituents might increase activity at one target while decreasing activity at others due to its bulky structure related to steric hindrance. 

Furthermore, extracts of *A. saligna* are selectively cytotoxic against cancerous cells while being non-toxic towards non-cancerous mammalian cells. The links between the biological effects, possible MAO, and SAR of the phytochemicals identified in *A. saligna* explain the observed bioactivities in the crude extracts and could help provide valuable insights towards future research and development of new therapeutics from this plant.

## Figures and Tables

**Table 1:** Taxonomy of *A. saligna*.

Domain	*Eurayota*
Kingdom	*Plantae*
Phylum	*Spermatophyta*
Subphylum	*Angiospermae*
Class	*Dicotyledonae*
Order	*Fabales*
Family	*Fabaceae*
Subfamily	*Mimosoideae*
Genus	*Acacia*
Species	*Acacia saligna* (Labill.) H. L. Wendl.

**Table 2:** Antioxidant activities of plant extracts in DPPH assay.

Plant Part/Origin	Type of Extract	IC_50_ µg/mL	Identified Compounds	Ref
Flowers/Tunisia	ethyl acetate	67.26	Isosalipurposide **1**, quercetin **3**, and naringenin **42**	[11]
Flowers/Egypt	Aqueous	461.71	Quercetin **3**, kaempferol **22**, naringenin **42,** benzoic acid **24**, syringic acid **28**, *p*-hydroxybenzoic acid **31**, salicylic acid **32**, caffeic acid **35**, *o*-coumaric acid **36**, *p*-coumaric acid **37**, ferulic acid **38**, ellagic acid **44**, catechol **45**, and caffeine **46**	[12]
Leaves/Saudi Arabia	methanolic	17.0	Quercetin **3**, rutin **6**, miquelianin **7**, isoquercetin **8**, hyperoside **9**, gallic acid **25**, and *p*-coumaric acid **37**	[13]
Bark/Egypt	Ethanolic	10.10	Quercetin **3**, rutin **6**, kaempferol **22**, benzoic acid **24**, gallic acid **25**, vanillin **29**, caffeic acid **35**, *o*-coumaric acid **36**, *p*-coumaric acid **37**, ferulic acid **38**, rosmarinic acid **39**, chlorogenic acid **40**, and caffeine **46**	[18]
Flowers/ Australia	methanolic	331.50	Naringenin **42,** isosalipurposide **1**, quercitrin **4**, *D-(+)*-pinitol **48**, and naringenin-7-*O*-*α*-*L*-arabinofuranoside **47**	[26]
Leaves/ Australia	methanolic	190.10	(–)-Epicatechin **51**, quercitrin **4**, myricitrin **11**, 2,4-di-*t*-butylphenol **50**, (−)-pinitol **49**, and (*3S,5S*)-3-hydroxy-5-(2-aminoethyl)-dihydrofuran-2(3H)-one **52**	[26]
Bark/ Australia	methanolic	94.24	(−)-Epicatechin **51**, *D-*(+)-pinitol **48**, and sucrose	[26]

**Table 3:** Antibacterial and antifungal extracts from *A. saligna*.

Plant Parts/Origin	Extracted Solvent	Bacteria	Fungi	Identified Compounds	Ref
Flowers/Egypt	water	*A. tumefaciens, E. cloacae*, *E. amylovora, P. carotovorum subsp*	*F. culmorum, R. solani, P. chrysogenum*	Quercetin **3**, kaempferol **22**, naringenin **42**, benzoic acid **24**, syringic acid **28**, *p*-hydroxybenzoic acid **31**, salicylic acid **32**, caffeic acid **35**, *o*-coumaric acid **36**, *p*-coumaric acid **37**, ferulic acid **38**, ellagic acid **44**, catechol **45**, and caffeine **46**	[12]
Leaves/Saudi Arabia	methanol	please refer to Table 4	please refer to Table 5	Quercetin **3**, rutin **6**, miquelianin **7**, isoquercetin **8**, hyperoside **9**, gallic acid **25**, and *p*-coumaric acid **37**	[13]
Leaves/Egypt	ethyl acetate	*S. aureus*, *S. pyogens, B.cereus, B. subtilius*	NR	Quercetin **3**, quercitrin **4**, quercetin-3-*O*-arabinoside **5**, myricetin **10**, myricitrin **11**, myricetin-3-*O*-arabinoside **12**, myricetin-3-*O*-glucoside **13**, (+)-catechin **14**, 7-*O*-Galloyl-cathecin **15**, apigenin **16**, apigetrin **17**, luteolin **19**, cynaroside **20**, gallic acid **25**, and methyl gallate **26**	[16]
Leaves/Saudi Arabia	ethanol	*E. coli*, *K. pneumonia*, *P. aeruginosa, Methicillin-resistant S. aureus (MRSA)*	*A. niger*, *A. fumigatus*, *A. flavus* and *C. albicans*	Gallic acid **25**, syringic acid **28**, chlorogenic **40**, *p*-hydroxybenzoic **31**, vanillic acid **29**, *p*-coumaric acid **37**, and salicylic acid **32**	[17]
Bark/Egypt	methanol	NR	*F. oxysporum*	Quercetin **3**, rutin **6**, kaempferol **22**, benzoic acid **24**, gallic acid **25**, vanillin **29**, caffeic acid **35**, *o*-coumaric acid **36**, *p*-coumaric acid **37**, ferulic acid **38**, rosmarinic acid **39**, chlorogenic acid **40**, and caffeine **46**	[18]

NR = not reported.

**Table 4:** Antibacterial activities (MIC and MBC) of methanolic leaf extract and identified pure compounds [13].

Extract and Compounds	*B. cereus*	*P. aeruginosa*	*L. monocytogenes*	*E. coli*	*M. flavus*	*S. aureus*
	MIC, MBC (mg/mL)	MIC, MBC (mg/mL)	MIC, MBC (mg/mL)	MIC, MBC (mg/mL)	MIC, MBC (mg/mL)	MIC, MBC (mg/mL)
*A. saliga* leaf extract	0.35, 0.73	0.37, 0.79	0.47, 0.99	0.31, 0.72	0.41, 0.85	0.30, 0.73
Rutin **6**	0.11, 0.22	0.07, 0.12	0.11, 0.21	0.12, 0.23	0.12, 0.23	0.1, 0.251
Hyperoside **9**	23.3, >500	27.2, >500	34.3, >500	31.2, >500	22.42, >500	19.54, >500
*p*-Coumaric acid **37**	0.12, 0.31	0.06, 0.22	0.26, 0.58	0.12, 0.25	0.16, 28.65	0.23, 0.47
Quercetin **3**	30.6, >500	31.8, >500	43.7, >500	38.6, >500	28.65, >500	21.53, >500
Miquelianin **7**	35.2, >500	25.3, 315.14	32.1, >500	31.8, >500	21.65, 411.17	17.1, 427
Streptomycin	0.07, 0.16	0.11, 0.21	0.12, 0.21	0.11, 0.20	0.10, 0.21	0.15, 0.31

**Table 5:** Antifungal activities (MIC and MBC) of methanolic leaf extract and identified pure compounds investigated by Elansary et al. [13].

Extract and Compounds	*Aspergillus* *flavus*	*Aspergillus* *ochraceus*	*L. monocytogenes*	*Candida* *albicans*	*Penicillium* *funiculosum*	*Penicillium* *ochrochloron*
	MIC, MBC (mg/mL)	MIC, MBC (mg/mL)	MIC, MBC (mg/mL)	MIC, MBC (mg/mL)	MIC, MBC (mg/mL)	MIC, MBC (mg/mL)
*A. saliga* extract	0.30, 0.91	0.38, 0.95	0.48, 1.02	0.58, 1.42	0.43, 1.01	0.44, 1.31
Rutin **6**	0.21, 0.45	0.18, 0.55	0.28, 0.62	0.25, 0.51	0.30, 0.71	0.23, 0.43
Hyperoside **9**	0.10, 0.46	0.13, 0.50	0.15, 0.52	0.21, 1.03	0.25, 1.03	0.31, 1.19
*p*-Coumaric acid	0.22, 0.43	0.23, 0.45	0.21, 0.41	0.32, 0.60	0.22, 0.59	0.20, 0.40
Quercetin **3**	0.31, 0.63	0.20, 0.75	0.21, 0.75	0.06, 0.33	0.24, 0.70	0.29, 0.63
Miquelianin **7**	0.26, 0.52	0.17, 0.61	0.18, 0.62	0.06, 0.27	0.21, 0.60	0.26, 0.54
Ketoconazole	0.20, 0.41	0.23, 0.46	0.10, 0.21	0.22, 0.43	2.05, 3.51	0.21, 0.40

**Table 6:** Inhibitory activities of extracts from flowers, leaves, and bark of Australian *A. saligna* and their isolated compounds and acarbose against yeast *α*-glucosidase [26].

Compound	IC_50_
Methanolic flower extract	34.93 μg/mL
Methanolic leaf extract	38.69 μg/mL
Methanolic bark extract	4.37 μg/mL
Isosalipurposide **1**	116.5 μM
Naringenin **42**	89.71 μM
Quercitrin **4**	177.3 μM
Myricitrin **11**	351.6 μM
Naringenin-7-O-α-*L*-arabinofuranose **47**	769.1 μM
*D*-(+)-pinitol **48**	74.69 μM
(–)-Pinitol **49**	164.2 μM
(–)-Epicatechin **51**	63.58 μM
2,4-Di-*t*-butylphenol **50**	259 μM
(3*S,*5*S*)-3-hydroxy-5-(2-aminoethyl) dihydrofuran-2(3*H*)-one **52**	>1000 ^a^
Acarbose	239.9 μM

^a^ The activity did not reach 50% at the highest tested concentration (1 mM).

## Data Availability

Not applicable.

## Supplementary Materials

The following supporting information can be downloaded at: https://www.mdpi.com/article/10.3390/molecules28114396/s1, Table S1. Summary of reported phytochemicals from various parts of *A. saligna*.

## Abbreviations

ABTS	2,2′-Azino-bis-(3-ethylbenzothiazoline-6-sulphonic acid)
Ag-NPs	Silver nanoparticles
AMPK	Adenosine-monophosphate-activated protein kinase
ATCC	American-type culture collection
BHT	Butylated hydroxytoluene
COX-2	Cyclooxygenase-2
DMSO	Dimethylsulfoxide
DCM	Dichloromethane
DNA	Deoxyribonucleic acid
DPPH	2,2-Diphenyl-1-picrylhydrazyl
DW	Dried weight
EtOAc	Ethyl acetate
EtOH	Ethanol
GC-FID	Gas chromatography with flame ionisation detection
GC-MS	Gas chromatography coupled with mass spectrometry
GLUT-4	Glucose transporter type 4
H. L. Wendl.	Heinrich Ludolph (Ludwig) Wendlan
HPLC	High-performance liquid chromatography
HPLC-DAD	HPLC–photodiode array detection
HPLC-VWD	HPLC-variable wavelength detector
IBD	Inflammatory bowel diseases
IC_50_	Half-maximal inhibitory concentration
IL-1b	Interleukin-1
LDL	Low-density lipid
MBC	Minimum bactericidal concentration
MFC	Minimum fungicidal concentration
MIC	Minimum inhibition concentration
MOA	Mechanism of action
MRSA	Methicillin-resistant *Staphylococcus aureus*
MTT	3-(4,5-Dimethylthiazol-2-yl)-2,5-diphenyl-tetrazolium bromide
NADH	Nicotinamide adenine dinucleotide H
NF-kB	Nuclear factor kappa B
NMR	Nuclear magnetic resonance
PGE_2_	Prostaglandin E2
PI3kt	phosphatidylinositol 3-kinase
Akt	serine/threonine kinase or serine/threonine kinase (PKB)
SAR	Structure-activity relationships
T2D	Type 2 diabetes
TEAC	Trolox equivalent antioxidant capacity
UC	Ulcerative colitis
VRE	Vancomycin-resistant enterococci
