# Supplementary material for: Bioactive Phytochemicals of Acacia saligna
Source: Molecules. 2023 May 28;28(11):4396. doi: 10.3390/molecules28114396 (PMC10254483; doi:10.3390/molecules28114396)
Supplement: Supplementary file 1 [file molecules-28-04396-s001.zip › molecules-2408751-supplementary.pdf]

Supplementary Information

Bioactive Phytochemicals of *Acacia saligna*

Alison T. Ung[1*] and Anjar P. Asmara[1]

[1] School of Mathematical and Physical Sciences, Faculty of Science, University of Technology Sydney, Ultimo, NSW 2007, Australia; anjarpurba.asmara@student.uts.edu.au

* Correspondence: alison.ung@uts.edu.au

Table S1. Summary of reported phytochemicals from various parts of *A. saligna*

Plant parts	Type of extract	Origin	Analytical methods	Identified compounds[1] (quantity)[c]	Ref[2]
Flowers	ethyl acetate	Tunisia	Single compound isolation[a] and structure elucidation[b]	*Quantity reported % w/w of dried flowers:* Isosalipurposide **1** (0.75), quercetin **3** (0.002), naringenin **42** (trace amount).	11
Flowers	aqueous	Egypt	HPLC	*Quantity reported in %w/w of crude extract:* Quercetin **3** (0.112), kaempferol **22** (0.0445), naringenin **42** (0.145), benzoic acid **24** (0.162), syringic acid **28** (0.006), *p*-hydroxybenzoic acid **31** (0.014), salicylic acid **32** (0.004), caffeic acid **35** (0.003), *o*-coumaric acid **36** (0.042), *p*-coumaric acid **37** (0.002), ferulic acid **38** (0.007), ellagic acid **44** (0.012), catechol **45** (0.007), and caffeine **46** (0.1)	12
Flowers	methanolic	Australia	Single compound isolation[a] and structure elucidation[b]	*Quantity reported in %w/w of dried flower***:** naringenin **42** (0.183)**,** quercitrin **4** (0.432), naringenin-7-O-α-*L*-arabinofuranoside **47** (0.27), D-(+)-pinitol 48 (0.262), isosalipurposide 1 (0.159)	26
Flowers	methanolic	Tunisia	GC-FID and GC-MS	*Content reported in % of total detected essential oils:* nonanal **56** (66.5% w/w), α-terpineol **57** (8.4%), heptadecan-2-one **58** (5.8%), tetradecanoic acid **59** (5%)	14
Leaves	methanolic	Saudi Arabia	HPLC	*Quantity reported in %w/w of crude extract:* Quercetin **3** (0.006), rutin **6** (1.533), miquelianin **7** (0.126), isoquercetin **8** (0.073), hyperoside **9** (0.633), gallic acid **25** (0.136), *p*-coumaric acid **37** (0.035).	13

Table S1. Summary of reported phytochemicals from various parts of *A. saligna*

Leaves	ethyl acetate	Egypt	Single compound isolation[a] and structure elucidation[b]	*Quantity reported in %w/w of crude extract:* Quercetin **3** (0.025), quercitrin **4** (0.030), quercetin-3-*O*-arabinoside **5** (0.030), myricetin **10** (0.025), myricitrin **11** (0.034), myricetin-3-*O*-arabinoside **12** (0.028), myricetin-3-*O*-glucopyranoside **13** (0.029), catechin **14** (0.025), 7-*O*-Galloyl-cathecin **15** (0.029), apigenin **16** (0.002), apigetrin **17** (0.002), luteolin **19** (0.029), cynaroside **20** (0.027), gallic acid **25** (0.027), methyl gallate **26** (0.030).	16
Leaves	methanolic	Saudi Arabia	HPLC	*Quantity reported in %w/w of crude extract:* gallic acid **25** (0.00543), syringic acid **28** (0.00037), chlorogenic **40** (trace), *p*-hydroxybenzoic **31** (0.0002), vanillic acid **29** (0.0002), *p*-coumaric acid **37** (0.00083), salicylic acid **32** (0.00013)	17
Leaves	methanolic	Egypt	Single compound isolation[a] and structure elucidation[b]	*Quantity reported in %w/w of dried leaves:* myricetin-3-O-rhamnoside (C7-O-C7) myricetin-3-O-rhamnoside **33** (0.001), quercitrin **4** (0.0023) and myricitrin **11** (0.00154), spirostane saponin (25*S*)-5*β*-spirostan-3*β*-yl-3-*O*-*β*-Dxylopyranosyl(1→3)-O-*β*-*D*-xylopyranosyl(1→4)-*β*-*D*-galactopyranoside **55** (0.0026), erythrodiol **53** (0.0081), 3*β*-*O*-trans-pcoumaroyl-erythrodiol **54** (0.00169)	25
Leaves	methanolic	Australia	Single compound isolation[a] and structure elucidation[b]	*Quantity reported in %w/w of dried leaves:* (–)-epicatechin **51** (0.0.91), quercitrin **4** (0.29), myricitrin **11** (0.507), 2,4-di-*t*-butylphenol **50** (0.101), (−)-pinitol **49** (0.913), (3*S*,5*S*)-3-hydroxy-5-(2-aminoethyl)-dihydrofuran-2(3H)-one **52** (0.507).	26
Leaves	methanolic	Tunisia	(GC-FID and GC/MS	*Content reported in % of total detected essential oils*: nonanal **56** (22.8%), *α*-terpineol **57** (12.1%), 4-oxoisophorone **60** (11.5%), linalool **61** (9.4%), heptadecan-2-one **58** (8.8%), and pentadecanal **62** (4.7%)	14
Leaves	ethanolic	Egypt	HPLC	*Quantity reported on % w/w of total phenolic content (140 µg /1g of dried leaves-GAE):* rutin **6** (15), naringin (7.5), taxifolin (4.6), (+)-catechin (50.8), vanillic acid **29** (1.8), syringic acid **28** (7.3), methyl gallate **26** (1.83), cinnamic acid (0.36), *p*-coumaric acid **37** (1.65), caffeic acid **35** (0.73), ferulic acid **38** (1.8), ellagic acid 44 (6.4).	30
Leaves	aqueous	Egypt	HPLC	*Quantity reported on % w/w of total phenolic content (200 µg /1g of dried leaves- GAE):* rutin 6 (23.2), naringin (5.2), quercetin 3 (3.2), (+)-catechin (23.6), gallic acid **25** (19.2), propyl gallate **27** (0.8), vanillic acid **29** (2), syringic acid **28** (1.2), cinnamic acid (0.32), *p*-coumaric acid **37** (6.4), chlorogenic acid **40** (12), ellagic acid **44** (2.8)	30

Table S1. Summary of reported phytochemicals from various parts of *A. saligna*

Bark	ethanolic	Egypt	HPLC	*Quantity reported in %w/w of crude extract:* Quercetin **3** (0.0037), rutin **6** (0.0016), kaempferol **22** (0.0011), benzoic acid **24** (0.0158), gallic acid **25** (0.0255), vanillin **29** (0.007), caffeic acid **35** (0.0054), *o*-coumaric acid **36** (0.0011), *p*-coumaric acid **37** (0.0008), ferulic acid **38** (0.0042), rosmarinic acid **39** (0.005), chlorogenic acid **40** (0.0104), and caffeine **46** (0.0107)	18
Bark	methanolic	Australia	Single compound isolation[a] and structure elucidation[b]	*Quantity reported in %w/w of dried barks:* (−)-epicatechin **51** (0.185), *D*-(+)-pinitol **48** (1.303), sucrose (6.87)	26
Seeds	methanolic	Tunisia	GC-MS	*Content reported in % of the total content of fatty acid (101.7 g/ kg of dried weight of seeds):* linoleic acid **63** (61.11–65.45% of total fatty acid content)**,** and oleic acid **64** (19.67–22.85%)**,** palmitic **65** (9.18–9.98%), stearic **66** (1.49–1.82%), vaccenic **67** (1.13–2.05%) and palmitoleic **68** (0.34–0.58%) acids	35

[a] compounds isolation by column chromatography; [b] structure elucidation by 1D & 2D NMR and mass spectrometry; [c] reported quantity of identified compounds.

[1] group of compounds colour code; green: flavonoids, red: benzoic acid and derivatives, blue: cinnamic acids, black: others.

[2]. Please refer to the references list in the main article.
